# Emotional Intelligence, Resilience, and Self-Esteem as Predictors of Satisfaction with Life in University Students

**DOI:** 10.3390/ijerph192416548

**Published:** 2022-12-09

**Authors:** Vilma Vilca-Pareja, Andrés Luque Ruiz de Somocurcio, Ronald Delgado-Morales, Lizbeth Medina Zeballos

**Affiliations:** 1 Departamento de Ciencias y Tecnologías Sociales y Humanidades, Facultad de Ciencias y Tecnologías Sociales y Humanidades, Universidad Católica de Santa María, Arequipa 04013, Peru; 2 Departamento de Psicología, Facultad de Ciencias y Tecnologías Sociales y Humanidades, Universidad Católica de Santa María, Arequipa 04013, Peru

**Keywords:** emotional intelligence, resilience, self-esteem, satisfaction with life, university students

## Abstract

The present study examined if Emotional Intelligence (EI), resilience, and self-esteem predicted life satisfaction in university students. We computed correlations between the study variables, then, we compared the differences between men and women. Finally, a simultaneous multiple regression was performed. The sample was composed of 2574 university students (715 were men and 1859 were women), whose age ranged from 18 to 30 years with a mean (M) of 20.83 and a standard deviation (SD) of 2.45. The instruments used were the Wong and Law Emotional Intelligence Scale (WLEIS), the Wagnild and Young Resilience Scale (ER-25), the Rosenberg Self-Esteem Scale (RSES), and the Diener Satisfaction with Life Scale (SWLS). The results indicated that EI, self-esteem, and resilience correlated significantly and directly with satisfaction with life. Regarding sex differences, it was found that men had greater resilience, appraisal and recognition of emotion in others, and self-regulation of emotion. Women had greater appraisal and expression of emotion in self and self-esteem. The results showed that self-esteem, self-regulation of emotion, the use of emotion to facilitate performance, and acceptance of self and life as resilience factors predicted satisfaction with life. accounting for 48% of the variance. The variable that best predicted satisfaction with life was self-esteem.

## 1. Introduction

There is a growing interest in investigating constructs framed within Positive Psychology as alternative solutions to promote mental health. Positive Psychology is an approach based on human strengths. This paradigm includes constructs such as emotional intelligence, resilience, self-esteem, subjective wellbeing, and optimism. Our study provides evidence about the predictive value of emotional intelligence, resilience, and self-esteem in life satisfaction in the Peruvian context. There is research on these variables in Europe and the USA; however, there is not enough evidence on how these variables predict satisfaction with life in the Peruvian and Latin American contexts.

Peru has its idiosyncrasies marked by maleness, a patriarchal culture, with high rates of family violence and violence against women, discrimination, and racism among other psychosocial problems. Therefore, it is important to investigate factors that could contribute to an increase psychological well-being in Peru.

### 1.1. Emotional Intelligence (EI)

EI, according to the Wong and Law model [1], uses Mayer and Salovey’s [2] conceptualization but also incorporates Gross’s model of emotional regulation [3]. EI is the ability to perceive, understand, and regulate one’s emotions and others [4,5,6]. Wong and Law [1] specify that EI is composed of four dimensions: (a) the ability to appraise and express emotions in self; (b) the ability to appraise and recognize emotion in others; (c) the ability to regulate one’s own emotions, which allows faster recovery from psychological discomfort; and (d) the use of emotion to facilitate performance, which is the ability of individuals to use their emotions to orient themselves towards constructive activities and personal performance.

We found two models developed for the EI: a model that conceives EI as a personality trait [7], and another that conceptualizes it as a capacity [2]. The latter is defined as the cognitive ability to process emotional information to resolve conflicts adaptively [8], while for Petrides [9], the Trait Emotional Intelligence (Trait EI) refers to people’s perceptions of their emotional abilities and is located at the lower levels of personality hierarchies.

EI is considered to moderate mental health’s adverse effects [10,11]. A higher EI is associated with greater satisfaction, job success [12,13], and better health [14]. EI correlates positively with self-efficacy in university students [15,16,17]. In addition, people with higher EI possess more developed social skills, are prosocial, less conflictive, and are better at coping with emotional difficulties [11,18]; people with a low level of EI are more likely to experience interpersonal difficulties and significant psychological problems [19].

### 1.2. Resilience

Resilience is the ability to show courage and adaptability when facing life’s misfortunes [20]. It is a positive trait that moderates the negative effects of stress and helps individuals adapt [21,22]. Wagnild and Young [22] mention that this variable comprises two factors: (a) personal competence and (b) acceptance of self and life. They also mention five characteristics of resilience: (a) meaningfulness, (b) existential aloneness, (c) self-reliance, (d) equanimity, and (e) perseverance.

### 1.3. Self-Esteem

Self-esteem is the attitude of acceptance or rejection of oneself [23,24]; it can be global or specific [25]. Global self-esteem is the individual’s positive or negative attitude toward the self as a totality [26,27]. This study measured global self-esteem, which is related to subjective wellbeing. Self-esteem is one of the factors that influences social functioning [28]. It is paramount to people’s success and wellbeing [29] and plays a protective role [30] against the effects of COVID-19 and contributes to one’s perceptions of their quality of life [31].

### 1.4. Satisfaction with Life (SWL)

Subjective wellbeing is a broad concept that includes experiencing high levels of pleasant emotions, low levels of negative emotions, and high satisfaction with life [32,33]. Therefore, subjective wellbeing is defined as a person’s global cognitive and affective assessments of their life [32,34,35]. Positive affects refers to pleasant emotions such as motivation, energy, desire for affiliation, achievement, or success; negative affects refers to unpleasant or uncomfortable emotions such as fear, inhibition, insecurity, frustration, and failure [36].

This study addressed the cognitive component of subjective wellbeing, i.e., SWL. SWL is a global judgment that people make about their life based on their unique criteria [37,38].

### 1.5. Emotional Intelligence, Resilience, Self-Esteem, and Satisfaction with Life

Meta-analytical studies, such as those performed by Sánchez-Álvarez et al. [39] and Xu et al. [40], showed that EI was associated with subjective wellbeing. There is research that shows that EI predicts SWL [6,18,41,42,43,44,45,46,47,48].

As for self-esteem, Liu and Fu [49], Pérez-Fuentes et al. [50], and Wang and Wu [51] showed that self-esteem correlated with SWL of university students. Self-esteem has been shown to predict SWL [52,53,54,55,56,57,58,59]. Moreover, Guasp et al. [60] used regression models to find that self-esteem and EI were significant predictors of SWL. Similarly, Arslan [61] found that self-esteem and resilience influenced SWL. Lacomba-Trejo et al. [62] showed that EI and resilience were associated with subjective wellbeing in their cognitive and affective components. As for resilience, it contributed positively to subjective wellbeing [63,64,65]. Salavera et al. [36] found that EI and self-esteem played an important role in wellbeing. Another study has corroborated the predictive capacity of EI and resilience for SWL [66].

### 1.6. Comparisons between Men and Women

Studies have shown that men generally have a better ability to manage and regulate emotions [67,68]. Mikolajczak et al. [68] showed that men scored higher on self-regulation of emotion, and women scored higher on appraisal and expression of emotion in self. D’Amico and Geraci [69] used the multi-trait and multi-method tool IE-ACCME (Intelligenza Emotiva: Abilitá, Credenze e Concetto di Sé Meta-Emotivo). This research showed that women scored higher than men on the appraisal and expression of emotion in self, suggesting a tendency in women to think and ruminate more about their own emotions, which in turn may trigger stress [41]. Ye et al. [70] found that women scored higher on self-esteem and SWL scores than men. Regarding resilience, Flórez and Sánchez [71] showed that men scored higher than women. Kumar [72] and Xie et al. [73] found that self-esteem was higher in men than in women. Finally, Gavín-Chocano et al. [8] found that men had higher scores on life satisfaction than women. Studies on sex differences in EI, resilience, self-esteem, and SWL are needed in Peru, where gender perspectives are different from Western cultures. Such studies may help develop programs to meet the needs of men and women in Peru.

The objectives of the present research were to (a) correlate EI, resilience, and self-esteem with SWL; (b) examine the differences between men and women based on the study variables; and (c) determine whether EI, resilience, and self-esteem predict SWL.

## 2. Method

### 2.1. Participants

The sample consisted of 2574 Peruvian students, 715 men and 1859 women from Arequipa, Peru, with a mean age of 20.83 years. They were recruited from two universities, one public and one private. To be included in the study, the students had to be over 18 years old and enrolled in Education, Psychology, Communication Sciences, or Social Work. All students gave their consent to participate in the study.

### 2.2. Procedure

The participants completed a sociodemographic sheet, followed by four scales measuring EI, resilience, self-esteem, and SWL. Data were gathered online using Google Forms. The students were recruited through school principals who authorized the researchers to enter hybrid classes at private and public universities through the meet platform. All students were given information about the research before requesting their consent to participate in this study. It was stressed that the responses were anonymous. This research was authorized by the Institutional Research Ethics Committee of the Universidad Católica de Santa María through resolution 015-22.

### 2.3. Instruments

#### 2.3.1. Wong and Law Emotional Intelligence Scale (WLEIS)

The Spanish version of WLEIS was validated by Extremera et al. [74] and adapted by Merino-Soto et al. [75] for studies in Peru. This instrument includes 4 dimensions and 16 items, with 4 items for each dimension [76]. The dimensions are: (a) appraisal and expression of emotion in self (item example: I have a good sense of why I have certain feelings most of the time); (b) appraisal and recognition of emotion in others (item example: I’m a good observer of others’ emotions); (c) self-regulation of emotion (item example: I am able to control my temper and handle difficulties rationally); and (d) the use of emotion to facilitate performance (item example: I always set goals for myself and then try my best to achieve them). It featured seven response alternatives on a Likert scale, ranging from 1 (completely disagree) to 7 (completely agree). Wong and Law [1] reported the Cronbach alpha reliability ranged from 0.83 to 0.90. In their research, Extremera et al. [75] reported the Cronbach alpha reliability: Self-Emotion Appraisal 0.79, Other’s Emotion Appraisal 0.81, Use of Emotion 0.81, and Regulation of Emotion 0.84.

#### 2.3.2. Resilience scale (ER-25)

The resilience scale is a self-report scale. It evaluates the degree of individual resilience through two factors: (a) Factor I, personal competence, composed of 17 items; (b) Factor II, acceptance of self and life, composed of 8 items. These factors represented the following characteristics of resilience: (a) meaningfulness, (b) existential aloneness, (c) self-reliance, (d) equanimity, and (e) perseverance [22]. This scale is composed of 25 items with a Likert scale of 7 points, ranging from 1 to 7. The sum of the scale scores is the total score, and the values range from 25 to 175. This scale was validated in Peru by Castilla et al. [77], and the Cronbach alpha reliability was 0.89. A representative item of this scale is “It’s okay if there are people who don’t like me”. Cejudo et al. [43] reported the Cronbach alpha reliability of 0.81.

#### 2.3.3. Rosenberg Self-Esteem Scale (RSES)

The RSES is a popular instrument used to evaluate perceived global self-esteem. This scale consists of 10 items, of which 5 are positively worded and 5 are negatively worded. In the rating of the scale, the negatively worded items are assigned an inverse score; for the overall score of self-esteem, the scores of all items are added together, allowing scores ranging between 10 and 40 points, where a higher score expresses high levels of self-esteem. It was adapted into Spanish by Martín-Albo et al. [78]. In Peru, it was adapted and validated by Ventura-León et al. [79], who reported the reliability of H >.80 (Index H is the measure of reliability, and it is interpreted in the same way as the Cronbach alpha reliability > 0.70). Ventura-León et al. [79] conducted a confirmatory factor analysis for validating RSES. In addition, for this study, we used the well-validated Spanish version. Pérez-Fuentes et al. [50] reported the Cronbach alpha reliability of 0.82. A representative item of this scale is “I believe that I have some good qualities”.

#### 2.3.4. Satisfaction with Life Scale (SWLS)

The SWLS measures the respondents’ perceptions of their satisfaction with life [80]. It consists of five items rated on a seven-point Likert scale. It was adapted into Spanish by Vázquez et al. [38], who obtained a Cronbach’s alpha of 0.87, and it was validated in Peru by Calderón de la Cruz et al. [81], who obtained a Cronbach’s alpha of 0.90. A representative item of this scale is “I am satisfied with my life”. Cejudo et al. [43] reported the Cronbach alpha reliability of 0.83.

### 2.4. Data Analysis

All data analyses were performed using the SPSS version 28 statistical program (IBM, 2016). The reliability of each instrument was examined by computing the Cronbach’s alpha coefficients (*α*) and descriptive statistics (M = mean; SD = standard deviation). Pearson’s r was computed between all variables. Then, independent sample *t*-tests were computed to examine sex differences, along with Cohen’s d values, which were evaluated by the following guidelines: <0.50 (small), 0.50–0.79 (moderate), and ≥0.80 (large). In addition, simultaneous multiple regression was used to examine how well the EI dimensions (appraisal and expression of emotion in self, appraisal and recognition of emotion in others, self-regulation of emotion, and the use of emotion to facilitate performance), resilience, and self-esteem predicted life satisfaction.

## 3. Results

The sample size was 2574 for all analyses. Table 1 presents the means and standard deviations of the study variables, as well as the evidence for reliability. It was evident that the instruments were reliable (above the 0.70 value).

Table 2 presents the correlations between self-esteem, resilience, and the EI dimensions (appraisal and expression of emotion in self, appraisal and recognition of emotion in others, self-regulation of emotion, and use of emotion to facilitate performance) that had a significant and direct correlation with SWL.

Sex differences were tested using the independent sample *t*-tests (see Table 3). The results show statistically significant differences in favor of men on resilience, appraisal and recognition of emotion in others, and self-regulation of emotion. The mean scores were significantly higher for women than men on self-esteem and appraisal and expression of emotion in self.

Simultaneous multiple regression analysis was performed. Self-esteem, resilience factors (personal competence and acceptance of self and life), and the EI dimensions (appraisal and expression of emotion in self, appraisal and recognition of emotion in others, self-regulation of emotion and use of emotion to facilitate performance) were considered predictor variables, and SWL was considered a criterion variable.

Table 4 presents the results of simultaneous multiple regression. The results revealed a coefficient of multiple determination of 0.48 indicating that self-esteem, self-regulation of emotion, the use of emotion to facilitate performance, and acceptance of self and life accounted for 48% of the variance in life satisfaction, while personal competence inversely predicted SWL. The variables appraisal and expression of emotion in self, and appraisal and recognition of emotion in others were not significant in SWL. To ensure the absence of multicollinearity, the tolerance values and the Variance Inflation Factor (VIF) were verified. As a rule of thumb, tolerance values < 0.10 and VIF > 10.0 are a sign of multicollinearity [82,83], the predictor variables did not have tolerance values < 0.10 nor VIF > 10.0.

## 4. Conclusions

The main objective of the present research was to determine whether EI, resilience, and self-esteem predicted SWL in the Peruvian context. We calculated the correlations between EI, resilience, self-esteem, and SWL. We also tested the differences between men and women.

All EI dimensions had significant and positive correlations with SWL. These results are consistent with Sánchez-Álvarez et al. [39] and Xu et al. [40], who also found these variables to be correlated. Resilience had a positive association with SWL, and these results were similar to those of Lacomba-Trejo et al. [62]. Self-esteem was positively associated with SWL. The studies by Holopainen et al. [55] and Rey et al. [58] support this result.

Differences in the mean scores between men and women were evident. Men scored higher on the appraisal and recognition of emotion, self-regulation of emotion, and resilience. We found that our results aligned with other research that found that men perceive themselves to be better at regulating their emotions. Women perceive themselves to be better at appraisal and expression of emotion (see [8,42,68]). We can interpret these results in light of the maleness and patriarchal culture implanted in Peru, where the social stereotypes established for men do not allow them to connect with their emotions; however, women are free to appraise and express their emotions.

The mean scores of self-esteem for women were higher than for men. The results were consistent with Ye et al. [70] who, in a study of college students, found that women had higher self-esteem scores than men. A possible explanation for the sex differences in self-esteem is that Peruvian women have had greater access to higher education in this millennium, giving them a higher sense of empowerment and self-confidence. Regarding the resilience variable, we found higher scores for men than for women; these results are similar to those in Flórez and Sánchez [71].

Self-regulation of emotion and use of emotion predicted SWL. It was not enough to evaluate, recognize, and express emotions to experience SWL. These results are consistent with Blasco-Belled et al. [41], Cejudo et al. [43], Extremera et al. [45], Kong et al. [46], Koydemir et al. [47], and Szcygiet and Mikolajczak [48]. Acceptance of self and life predicted SWL; these results coincide with those found by Hartson et al. [63], Rasheed et al. [64], and Zhao et al. [65]; however, personal competence negatively predicted SWL. Self-esteem was the best predictor of SWL, and these results were similar to those of other studies (see [50,52,57,59]). Self-esteem, self-regulation of emotion, use of emotion, and the acceptance of self and life jointly predicted 48% of the variance in SWL, a high percentage when explaining the factors that predict SWL. We postulate that when individuals have the ability to process emotions and feel good about themselves, they are more likely to experience wellbeing. On the contrary, individuals who do not adequately regulate their emotions and have difficulty properly using their emotions experiences unhappiness and probably generates discomfort around them.

The implications of the findings relate to the need to design and implement emotional education programs that involve issues such as self-esteem, resilience, and EI to increase SWL in university students. This study contributes to understanding possible predictors of SWL in the Peruvian-Latin American context in university students.

A limitation of this study was that we used a sample of university students, which do not allow us to generalize the results to other populations such as children, adolescents, and adults. In addition, the sample was primarily made up of women, which could affect the results. We suggest equating the numbers of men and women in the sample in future studies. It is necessary to replicate this research with students pursuing other professional careers to analyze the behavior of these variables. Finally, we suggest including the affective component of subjective wellbeing in future studies.

## Figures and Tables

**Table 1:** Descriptive statistics and reliability.

	M	SD	*α*
Appraisal and expression of emotion in self	22.10	4.00	0.70
Appraisal and recognition of emotion in others	22.80	3.40	0.78
Self-regulation of emotion	20.50	4.40	0.78
Use of emotion to facilitate performance	21.80	4.10	0.71
Resilience	128.50	27.70	0.96
Self-esteem	2.80	0.50	0.86
Life satisfaction	23.50	6.50	0.89

M: mean; SD: standard deviation; *α*: Cronbach’s alpha.

**Table 2:** Correlation matrix of the study variables (n = 2574).

	1	2	3	4	5	6	7
1. Self-esteem	–						
2. Resilience	0.40 **	–					
3. Appraisal and expression of emotion in self	0.51 **	0.27 **	–				
4. Appraisal and recognition of emotion in others	0.26 **	0.18 **	0.54 **	–			
5. Use of emotion to facilitate performance	0.62 **	0.33 **	0.65 **	0.51 **	–		
6. Self-regulation of emotion	0.48 **	0.24 **	0.71 **	0.43 **	0.62 **	–	–
7. Life satisfaction	0.63 **	0.24 **	0.48 **	0.29 **	0.57 **	0.48 **	–

** *p* < 0.001.

**Table 3:** Sex differences.

	Men (715)		Women (1859)		*t*		
Variables	M	SD	M	SD	(2572)	*p*	d
Self-esteem	126.30	32.00	129.40	25.80	2.94	0.003	0.13
Resilience	22.70	3.90	21.90	4.00	−2.55	0.011	−0.11
Appraisal and expression of emotion in self	22.60	3.50	22.90	3.30	4.81	0.001	0.21
Appraisal and recognition of emotion in others	22.00	4.00	21.70	4.10	−1.99	0.046	−0.09
Self-regulation of emotion	2.90	0.50	2.80	0.50	5.56	0.001	0.24
Use of emotion to facilitate performance	21.30	4.30	20.20	4.40	1.42	0.155	0.06
Life satisfaction	23.90	6.60	23.40	6.40	1.65	0.098	0.07

M: mean; SD: standard deviation; *t*= independent samples *t*-test; d: effect size using Cohen d.

**Table 4:** Simultaneous multiple regression analysis to predict satisfaction with life.

Variables	Dimensions	Predictors				
		B	R^2^	*β*	*t*	*p*
			0.48			
Self-esteem	Global self-esteem	5.08		0.41	21.04	0.001
Resilience	Personal competence	−0.11		−0.32	−9.41	0.001
	Acceptance of self and life	0.21		0.29	8.35	0.001
Emotional Intelligence	Appraisal and expression of emotion in self	0.05		0.03	1.43	0.154
	Appraisal and recognition of emotion in others	0.04		0.02	1.04	0.300
	Self-regulation of emotion	0.17		0.12	5.45	0.001
	Use of emotion to facilitate performance	0.35		0.22	9.80	0.001

B: non-standardized beta coefficient; *β* standardized beta coefficient.

## Data Availability

The data presented in this study are available on request from the corresponding author. The data are not publicly available due to we also require the authorization of the Vice-Rectorate for Research of Universidad Católica de Santa María.
